# Antibacterial and Antibiofilm Properties of the Alexidine Dihydrochloride (MMV396785) against *Acinetobacter baumannii*

**DOI:** 10.3390/antibiotics12071155

**Published:** 2023-07-06

**Authors:** Kirti Upmanyu, Qazi Mohd. Rizwanul Haq, Ruchi Singh

**Affiliations:** 1 ICMR-National Institute of Pathology, Safdarjung Hospital Campus, New Delhi 110029, India; kirtiupmanyu@gmail.com; 2 Department of Biosciences, Jamia Millia Islamia, A Central University, New Delhi 110025, India; qhaque@jmi.ac.in

**Keywords:** biofilm, antibacterial, *Acinetobacter baumannii*, alexidine dihydrochloride, biofilm inhibition, biofilm eradication

## Abstract

Antibiotic-resistant *Acinetobacter baumannii* infections among patients in hospital settings are rising at an alarming rate. The World Health Organization has designated carbapenem-resistant *A. baumannii* as a priority pathogen for drug discovery. Based on the open drug discovery approach, we screened 400 compounds provided as a Pandemic Response Box by MMV and DNDi to identify compounds with antibacterial and antibiofilm activity against two *A. baumannii* reference strains using a highly robust resazurin assay. In vitro screening identified thirty compounds with MIC ≤ 50μM having growth inhibitory properties against the planktonic state. Five compounds, with MMV IDs MMV396785, MMV1578568, MMV1578574, MMV1578564, and MMV1579850, were able to reduce metabolically active cells in the biofilm state. Of these five compounds, MMV396785 showed potential antibacterial and antibiofilm activity with MIC, MBIC, and MBEC of 3.125 μM, 12.5, and 25–100 µM against tested *A. baumannii* strains, respectively, showing biofilm formation inhibition by 93% and eradication of pre-formed biofilms by 60–77.4%. In addition, MMV396785 showed a drastic reduction in the surface area and thickness of biofilms. Further investigations at the molecular level by qRT-PCR revealed the downregulation of biofilm-associated genes when exposed to 50 µM MMV396785 in all tested strains. This study identified the novel compound MMV396785 as showing potential in vitro antibacterial and antibiofilm efficacy against *A. baumannii*.

## 1. Introduction

The growing burden of antimicrobial-resistant ESKAPE (Enterococcus faecium, Staphylococcus aureus, Klebsiella pneumoniae, *Acinetobacter baumannii*, Pseudomonas aeruginosa, and Enterobacter species) pathogens is a major global concern for public health [1,2,3]. Amongst these, *Acinetobacter baumannii*, a Gram-negative coccobacillus, has predominantly emerged as a “superbug” by rapidly developing resistance to multiple structurally distinct antibiotics. In addition, *A. baumannii* is associated with nosocomial infections among immune-compromised individuals, especially those admitted to intensive care units (ICUs) [4]. Gradually, it has also become a culprit in community-acquired infections [5]. High mortality rates of 35–60% among patients with *A. baumannii* infections have been reported [6,7,8,9]. In addition, comorbid disorders, prolonged hospitalization, and nosocomial infections pose high mortality risks [10]. Such high fatality rates and lack of treatment options have led WHO to classify carbapenem-resistant *A. baumannii* as the “priority pathogen” to accelerate the development of new antimicrobials for treatment.

*A. baumannii* can colonize and form biofilms on clinically relevant substances and biotic surfaces, which is the root cause of its spread and persistence in the hospital environment. Biofilms are structured bacterial communities attached to a surface by secreting extracellular polymeric substances, such as polysaccharides, proteins, lipids, and nucleic acids, constituting an impermeable biofilm matrix hiding bacterial cells within it. A diverse range of biofilm-associated *A. baumannii* infections, including ventilator-associated pneumonia, bacteremia, meningitis, and catheter-associated urinary tract infection can severely impede the health of critically ill patients in ICUs [11,12,13].

Colonization of indwelling devices by pathogens provides a mode of entering the body of patients admitted to hospitals. The development of biofilms is a multifactorial process involving adhesins such as a chaperone-usher pili system encoded by the *csu* operon, and outer membrane proteins OmpA and Omp33. Polysaccharides such as poly-N-acetyl glucosamine (PNAG) synthesized by pga operon and alginate produced by the bi-functional protein phosphomannomutase/phosphoglucomutase encoded by the *algC* gene are major components of the biofilm matrix. Proteins such as biofilm-associated protein (Bap) and amyloidogenic proteins are responsible for constructing matured three-dimensional structures of biofilms. Various regulatory mechanisms include a two-component system BfmRS, c-di-GMP, and quorum sensing. Controlling *A. baumannii* infections involves targeting these factors to eradicate the pre-formed biofilms caused by these factors [14].

Infections associated with biofilms pose a challenge in treatment with the current antibiotics because the impermeable matrix limits the amount of antibiotic reaching bacterial cells [15,16]. Biofilm formation has, therefore, contributed to the pathogenicity of this bacterium. Conventional antibiotics such as colistin, ciprofloxacin, and imipenem, even at a thousand times higher than bactericidal concentrations for cells growing in planktonic conditions, were insufficient to eradicate strong *A. baumannii* biofilms [17]. Due to the high antibiotic tolerance of biofilms, monotherapy is considered inappropriate, and combination therapy with currently available antibiotics only inhibits biofilm formation but is inefficient in eradicating pre-formed biofilms [18]. To stimulate the production of antibiotics for treating *A. baumannii* infection, in this study, we screened for compounds with antibacterial and antibiofilm effects against *A. baumannii*. An open-source library of chemically synthesized compounds assembled as a Pandemic Response Box by the Medicines for Malaria Venture (MMV) and Drugs for Neglected Diseases initiative (DNDi) was used in this study. The MMV Pandemic Response Box contains 402 compounds, of which 201 are categorized as antibacterial, 46 are antifungal, and 153 are antiviral. On screening compounds from the pandemic box, we identified MMV396785 (Alexidine dihydrochloride (ADH)), classified as an antifungal compound, as showing potential antibacterial and antibiofilm effects against *A. baumannii*.

## 2. Material and Methods

### 2.1. Bacterial Isolates, Reagents, and Culturing Conditions

Standard *Acinetobacter baumannii* strains of clinical origin AB-BC-5 and 3-137 obtained from BEI resources (Biodefence and Emerging Infections Research Resources Repository), ATCC 19606 (Himedia), and an environmental isolate HK-45 were used in this study. Cultures were grown in cation-adjusted Mueller Hinton broth (CAMHB) for screening antimicrobial compounds and tryptic soy broth for biofilm assay [19,20]. The open-source Pandemic Response Box was made available by Medicine for Malaria Venture (MMV), and Alexidine dihydrochloride (ADH) was procured from Sigma.

### 2.2. Screening Compounds from the Pandemic Box against A. baumannii

The MMV Pandemic Response Box contained 10 mM of each of the 400 compounds, which was further diluted to 1 mM using DMSO. Antimicrobial susceptibility was determined using resazurin redox reagent, which changes color from blue to pink (resorufin dye) on reduction by metabolically active cells, as described previously [21,22]. Isolates BC-5 and ATCC 19606 were grown till the OD_600_ reached 0.1. Then, cultures were further diluted 1:100, and 10 μL aliquots of the diluted cultures were added to a 96-well plate containing compounds ranging in concentration from 50 μM to 0.78 μM. A positive control containing bacterial inoculum without any drug (2.5% *v*/*v* DMSO) and a negative control or blank without any inoculum, along with two internal controls, colistin and chloramphenicol with known MIC, were used each time. The plate was kept at 37 °C with shaking at 220 rpm overnight. OD_600_ was recorded after incubation for 16–18 h at 37 °C. Then, 20 μL of resazurin (0.015% *w*/*v*) was added to all the wells and kept for another 1–2 h at 37 °C with shaking. The change in color from blue to pink indicated bacterial growth; therefore, the lowest concentration of antibiotic at which a color change was not observed was considered as the MIC of that compound.

### 2.3. Time–Kill Assay

Time–kill kinetics assays of ADH were conducted against ATCC 19606, BC-5, 3-137, and HK-45, as described earlier [23]. A bacterial inoculum of 1 × 10^6^ cfu/mL (OD_600_ = 0.1) of each isolate was allowed to grow in the presence of ADH at 2 × MIC (6.25 μM), 1 × MIC (3.125 μM), 1/2 × MIC (1.56 μM), and 1/4 × MIC (0.78 μM). The cultures were serially diluted accordingly and plated on Luria agar plates at 0 h, 2 h, 4 h, 8 h, and 24 h. Colonies were counted after 16–18 h of incubation to calculate viable cfu/mL.

### 2.4. Biofilm Formation Inhibition

The ability of ADH to inhibit *A. baumannii* biofilm formation on polystyrene was determined using a semi-quantitative crystal violet assay, as described previously [24]. The bacterial cells were cultured in TSB containing 2.5 g/L of glucose till the OD_600_ reached 0.1. Further, cultures were diluted in a 1:1 ratio in fresh TSB medium. An amount of 100 μL of TSB containing ADH at concentrations ranging from 0 to 50 μM and 100 μL of diluted culture was added to a 96-well plate and kept at 37 °C for 48 h without shaking. After incubation, planktonic cells from wells were discarded, and wells were washed thrice with 200 μL of 1× PBS. An amount of 200 μL of 0.1% crystal violet was added to the wells and incubated for 30 min at RT to stain the biofilms. Cells that adhered to the wells were stained with 200 μL of 0.1% crystal violet and incubated at room temperature for 20 min. The wells were rewashed three times with water and air-dried for 20 min. An amount of 200 μL of 95% (*v*/*v*) ethanol was added to solubilize the stained biofilms, followed by measuring the absorbance at 570 nm in an ELISA plate reader (TECAN Infinite M200, Switzerland). The results of three individual experiments performed in triplicate resulting in nine measurement values were averaged (*n* = 3 × 3).

### 2.5. Biofilm Eradication Assay

A biofilm eradication assay was performed to assess the antibiofilm property of compounds on pre-formed biofilms and to determine the minimum microbial biofilm eradication concentration (MBEC) of ADH [24]. Biofilms were allowed to form in a 96-well plate as mentioned above, without adding any compound. After 48 h of incubation, the cultures were removed, and 200 μL of fresh TSB-containing 50 μM compound to be assessed from the pandemic box or 0–100 μM ADH was added to the wells and incubated for another 24 h at 37 °C under static conditions. After 24 h, the planktonic cells were discarded, and the wells were rinsed thrice with 200 μL of 1 × PBS. The adhered biomass was stained with 0.1% crystal violet and incubated for 30 min at RT. Then, the dye was removed, and the wells were washed thrice with distilled water. The stained biomass was dissolved in 95% ethanol, and absorbance was recorded at 570 nm in an ELISA plate reader. The biofilm eradication percentage was calculated compared to the biofilm formed without antibiotic exposure (defined as 100%) and the TSB-only control (defined as 0%).

### 2.6. Confocal Laser Scanning Microscopy

Biofilms were allowed to form on the glass coverslip by bacterial cells for 48 h, and were exposed to TSB containing 0 μM, 25 μM, and 100 μM ADH for 24 h. Then, coverslips were washed three times with filtered Milli-Q water, and biofilms were stained with a LIVE/DEAD BacLight Bacterial viability kit (L13152, Invitrogen, Waltham, MA, USA) containing fluorescent probes Syto 9 and propidium iodide (PI) at a concentration of 3 µL/mL, as mentioned previously [25]. Further, the biofilms were allowed to stain for 30 min in the dark, and then excess dye was removed by washing with filtered Milli-Q water. Images were acquired using inverted confocal laser scanning microscopy (UC7, Leica Microsystems, Wetzlar, Germany). The images were obtained at a magnification of 400×. Z-stacks were attained along the thickness with the scale of 1 µm.

### 2.7. RNA Isolation and cDNA Preparation

Bacterial cells at a final OD_600_ of 0.025 and 4 mL culture volume were allowed to grow in a 12-well plate for biofilm formation as described previously, with modifications [26]. After 48 h of incubation, planktonic cells were removed, and 4 mL TSB media supplemented with 0 μM (untreated control), 25 μM (8 x MIC of ADH), and 50 μM (16 x MIC of ADH) ADH was added. The plate was further incubated for 24 h. After incubation, the biofilm-associated cells were scraped and re-suspended in the media. RNA was extracted using a GF-1 total RNA extraction kit (Vivantis, Malaysia) as per the manufacturer’s instructions. cDNA was synthesized using a Protoscript II First Strand cDNA synthesis kit (New England Biolabs, USA) as per the manufacturer’s instructions. The prepared cDNA was stored at −20 °C until further use.

### 2.8. Quantitative Real-Time PCR Assay

The expression of five biofilm-associated genes (ompA, csuE, abaI, pgaC, and bfmR) was analyzed using the primers listed in Table 1. 16SrRNA and rpoB were used as the internal controls. A qRT-PCR assay was performed to analyze the changes in the expression of these virulence genes on exposure to ADH. A 10 μL reaction was set up comprising 5 μL of Fast SYBR green master mix (Applied Biosystems™ 4385612, USA), 1 μM of each gene-specific forward and reverse primer, cDNA template, and nuclease-free water to make up the volume to 10 μL. The reaction was performed using a CFX96 Real-Time PCR System (Bio-Rad, USA) under the following conditions: 95 °C for 15 min, followed by 40 cycles of 95 °C for 15 s, annealing for 30 s at 55 °C, and 72 °C for 30 s. The quantity of gene-specific amplicons was calibrated with 16S rRNA as internal controls. The relative gene expression analysis was calculated using the 2^−∆∆Ct^ method.

### 2.9. Checkerboard Assay

Checkerboard assays were carried out to test the MIC of tigecycline, rifampicin, trimethoprim, and colistin in combination with ADH, as described previously [27]. In brief, decreasing concentrations of ADH (64–0.125 μg/mL) in each column and decreasing concentrations of antibiotic (32–0.5 μg/mL) in each row were set up in 96-well microtiter plates. An amount of 100 μL of Mueller–Hinton broth (MHB) was added to all the wells. An amount of 100 μL of media containing four times the highest required concentration of antibiotic was added in the first row. The antibiotic was serially diluted in each row. Similarly, 100 μL of media containing twice the highest required concentration of ADH was added in the first column and was serially diluted in each column. Each well was inoculated with 10 μL of 1:100 diluted culture of the test strain with 0.1 OD_600_. The plates were incubated at 37 °C on an incubator shaker for 24 h. After incubation, cell growth was quantified by measuring the absorbance at 600 nm. Fractional inhibitory concentration was calculated as below, and the results were interpreted as follows: synergistic (FICi ≤ 0.5), additive (0.5 < FICi ≤ 1), indifferent (1 < FICi ≤ 4), and antagonistic (FICi > 4) [23,27].

### 2.10. Statistical Analysis

Each assay was performed at least twice, MBIC and MBEC were performed in triplicates (*n*= 6–9), and the results below are shown as mean ± standard deviation. The treated and untreated controls were compared using a one-way ANOVA analysis of variance followed by Dunnett’s test. A two-tailed *t*-test was used to analyze biofilm-associated cfu in the control and the treated wells. Z-factor was calculated as described previously [21,28]. GraphPad Prism version 5.1 was used for all the statistical analyses and plotting graphs.

## 3. Results

### 3.1. Antibacterial Compounds Identified against A. baumannii from the MMV Pandemic Response Box

**Figure 1:**
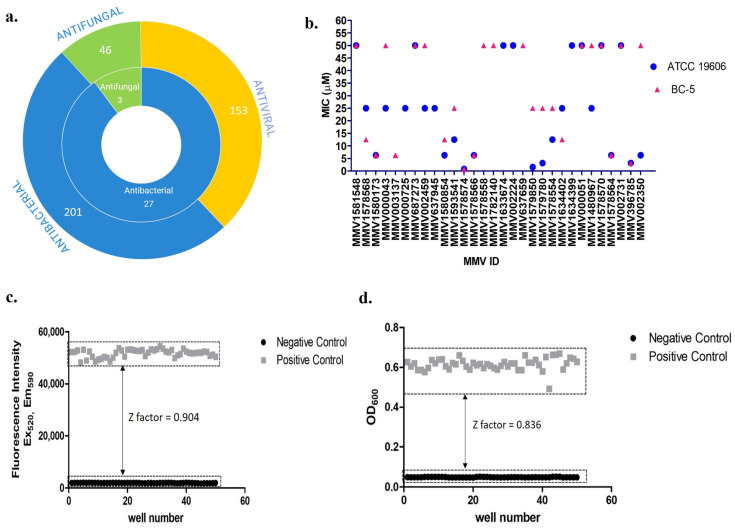
(**a**) MMV pandemic box screening identified 30 compounds (3 antifungal and 27 antibacterial) with antimicrobial activity against *A. baumannii*. The outer circle represents the total compounds classified as antibacterial, antifungal, and antiviral according to MMV. The inner circle represents antimicrobial compound hits, with MIC ≤ 50 µM. (**b**) MICs of 30 identified antimicrobial compounds against *A. baumannii* isolates ATCC 19606 and BC-5. (**c**) Validation of resazurin-based screening assay demonstrating a Z-factor of 0.904 in comparison with (**d**) absorbance-based assay with a Z-factor of 0.836, for high throughput screening against *A. baumannii*.

A total of 400 compounds from the MMV Pandemic Response Box were screened for their antibacterial activity against 2 different *A. baumannii* strains, BC-5 and ATCC 19606. Of these 400 compounds, 30 compounds (7.5%) exhibited growth inhibition at a minimum inhibitory concentration (MIC) ≤ 50 μM against *A. baumannii* ATCC 19606 or BC-5, or both (Figure 1, Supplementary Table S1). Twenty-seven (90%) of these thirty compounds were antibacterials, and three were antifungal as per MMV classification (Figure 1a.). Nine of these thirty compounds showed growth inhibition at ≥25 μM against both ATCC 19606 and BC-5. Another batch of nine compounds could inhibit the growth of either ATCC 19606 or BC-5, but not both. Only twelve (40%) of these thirty compounds inhibited the growth of ATCC 19606 and BC-5 at ≤25 μM, and were further screened for their antibiofilm efficacy against *A. baumannii* (Figure 1b).

The Z-factor that determines the robustness and reliability of the assay was calculated by the degree of separation between the positive (drug-free media and 2.5% DMSO) and the negative controls (culture-free media and 2.5% DMSO) for the resazurin assay and compared with the measurements of absorbance at 600 nm. The fluorescence was measured 4 h after the addition of resazurin with excitation at 520 nm and emission at 590 nm. The Z-factor obtained for the resazurin assay was 0.904, and that of the OD_600_ measurements was 0.83, demonstrating the reliability and robustness of the method adopted for investigating the activity of compounds by screening pandemic box compounds.

### 3.2. Antibiofilm Efficacy of Compounds with Antibacterial Activity against A. baumannii

**Figure 2:**
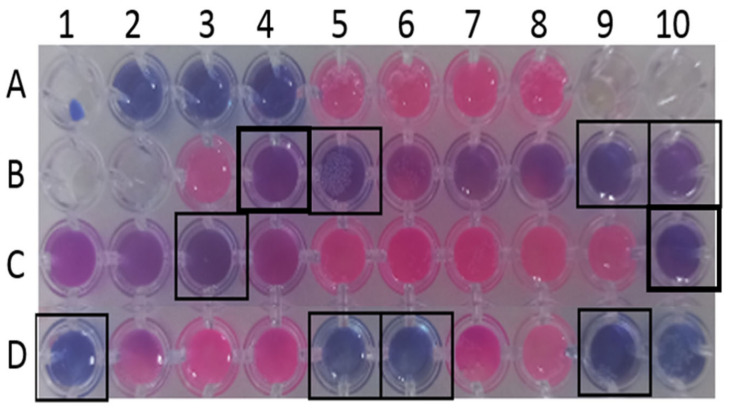
Metabolic activity of bacterial cells analyzed by resazurin post-biofilm-treatment with compounds from the Pandemic Response Box: negative control (A2–A4); 19606 control (A5–A8); 19606 treated (B3–C4) MMV1580173 (B3), MMV396785 (B4), MMV1578568 (B5), MMV1578554 (B6), MMV1634402 (B7), MMV1579780 (B8), MMV1579850 (B9), MMV1578564 (B10), MMV1593541 (C1), MMV1580854 (C2), MMV1578574 (C3), MMV1578566 (C4), BC-5 control (C5–C8), BC-5 treated (C9–D10) MMV1580173 (C9), MMV396785 (C10), MMV1578568 (D1), MMV1578554 (D2), MMV1634402 (D3), MMV1579780 (D4), MMV1579850 (D5), MMV1578564 (D6), MMV1593541 (D7), MMV1580854 (D8), MMV1578574 (D9), and MMV1578566 (D10).

**Figure 3:**
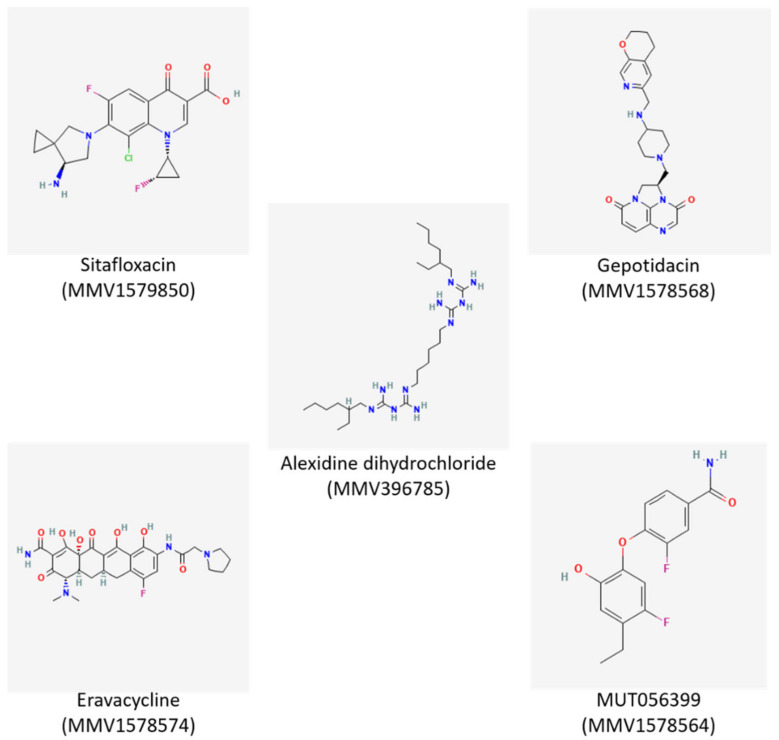
Structures of five antibiofilm compounds identified against *A. baumannii* biofilms.

A total of 12 of these 30 pandemic box compounds with MIC ≤ 25 µM against both ATCC 19606 and BC-5 *A. baumannii* isolates were analyzed for their ability to eliminate *A. baumannii* growth in the biofilm state. Five of the above twelve compounds, alexidine dihydrochloride (MMV396785), gepotidacin (MMV1578568), eravacycline (MMV1578574), MUT056399 (MMV1578564), and sitafloxacin (MMV1579850) at 50 µM were able to reduce metabolically active cells and could eradicate pre-formed biofilms (Figure 2, Supplementary Figure S1). The chemical structures and trivial names of the compounds identified as antibiofilm inhibitors against *A. baumannii* are shown in Figure 3.

Of the five compounds with antibiofilm properties against *A. baumannii* 19606 and BC-5, alexidine dihydrochloride (ADH) was selected for further analysis due to its low minimum inhibitory concentration (MIC) value of 3.125 µM and wide range of actions.

### 3.3. ADH Is Bactericidal for A. baumannii

**Figure 4:**
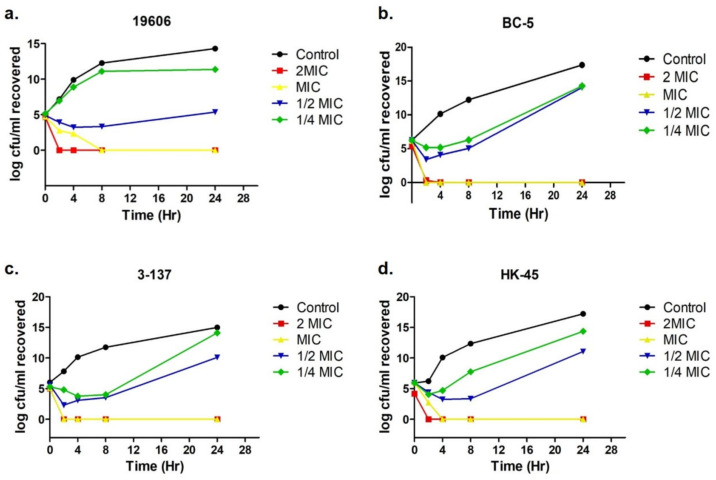
Time–kill assays of (**a**) ATCC 19606, (**b**) BC-5, (**c**) 3-137, and (**d**) HK-45 in the absence and presence of 2 × MIC, MIC, ½ × MIC, and ¼ × MIC of ADH.

The pharmacodynamics of ADH against four *A. baumannii* isolates, ATCC 19606, BC-5, 3-137, and HK45, was examined using a time–kill assay. The curves obtained from the time–kill assay revealed that ADH rapidly reduced *A. baumannii* numbers, resulting in >5 log10 cfu/mL reduction within 2 h due to its bactericidal activity at 2 × MIC (6.25 µM) against all the tested isolates (Figure 4). ADH at MIC (3.125 µM) could completely eradicate BC-5 and 3-137 within 2 h (Figure 4b,c); however, for 19606 and HK45, complete bactericidal effects at the MIC were observed at 8 and 4 h, respectively (Figure 4a,d).

### 3.4. Antibiofilm Effects of ADH on Biofilm Formation by A. baumannii

**Figure 5:**
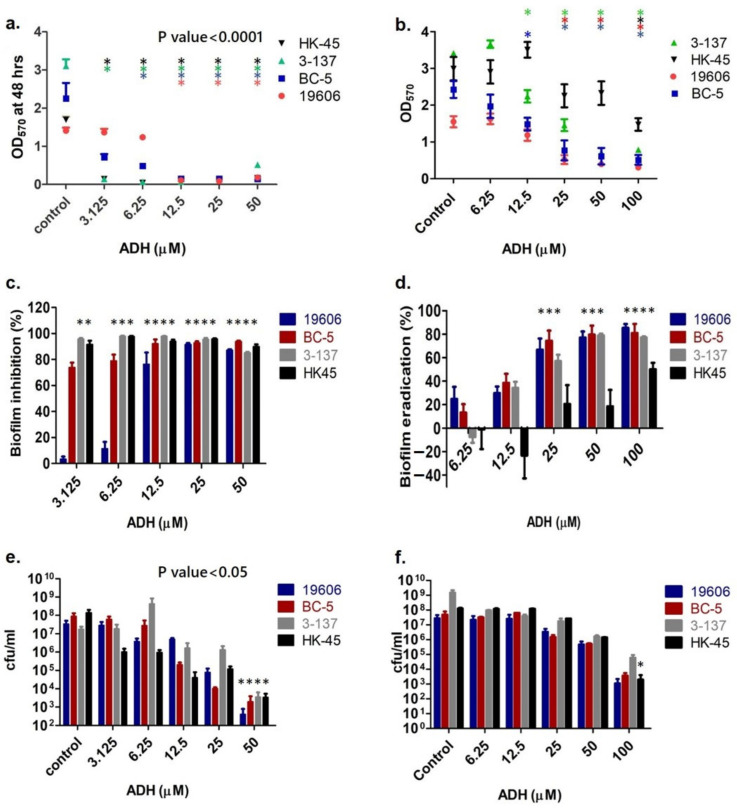
Crystal violet staining of biofilm formed by *A. baumannii* isolates ATCC 19606, BC-5, 3-137, HK-45 in the absence or presence of ADH at concentrations ranging from 3.125 to 100 μM for determining (**a**) microbial biofilm inhibition concentration and (**b**) microbial biofilm eradication concentration. (**c**) Percentage biofilm inhibition and (**d**) eradication by ADH for *A. baumannii* isolates. Biofilm-associated viable bacterial cells in the control and when treated with ADH (**e**) prior to biofilm formation and (**f**) post-biofilm formation. *, *, *, * *p* value < 0.0001 when compared with control for HK-45, 3-137, BC-5 and 19606, respectively (for **a**–**d**). * *p* value < 0.05 as compared to control (for **e** and **f**).

The potential of ADH to inhibit biofilm formation by *A. baumannii* strains ATCC 19606, BC-5, 3-137, and HK45 was investigated in 96-well polystyrene microtiter plates using a crystal violet assay. Wells treated with ADH showed significantly reduced or no biofilm formation in a concentration-dependent manner (*p* < 0.0001). ADH also successfully inhibited biofilm formation by strong biofilm-forming isolate 3-137 at the MIC (3.125 μM). At 12.5 μM (4 × MIC), ADH inhibited more than 90% of biofilm formation by all the isolates, including strong, weak, and moderate biofilm formers (Figure 5a,c). Further, determining the colony-forming units to analyze the viable bacterial cells on treatment with ADH showed approximately a 4-fold log10 reduction in biofilm-associated bacterial cell count in all four isolates when treated with ADH at a concentration of 50 μM as compared to controls (Figure 5e).

The ability of ADH to eradicate pre-formed *A. baumannii* biofilms was also analyzed using a crystal violet assay (Figure 5b). On treating 48 h old biofilms with ADH at 6.25 μM (2 × MIC) and 12.5 μM (4 × MIC) concentrations, a slight increase in biofilm formation was observed in the case of ATCC 19606, 3-137, and HK-45, respectively (Figure 5b,d). However, ADH at 25 μM (8 × MIC) concentration significantly reduced the biomass of biofilms formed by ATCC 19606, BC-5, and 3-137 by more than 60%. At a higher concentration of 100 μM, the biomass of the biofilms formed by all four isolates was significantly reduced (Figure 5d). Most antibiotics are known to promote biofilm formation and are required at 1000 times MIC to eradicate pre-formed biofilms [29]. On further calculating the number of viable biofilm-associated cells, it was found that ADH could significantly reduce the cfu by ≥4 log_10_ fold at a concentration of 100 μM (Figure 5f).

### 3.5. Confocal Laser Scanning Microscopy Shows Biofilm Eradication by ADH

**Figure 6:**
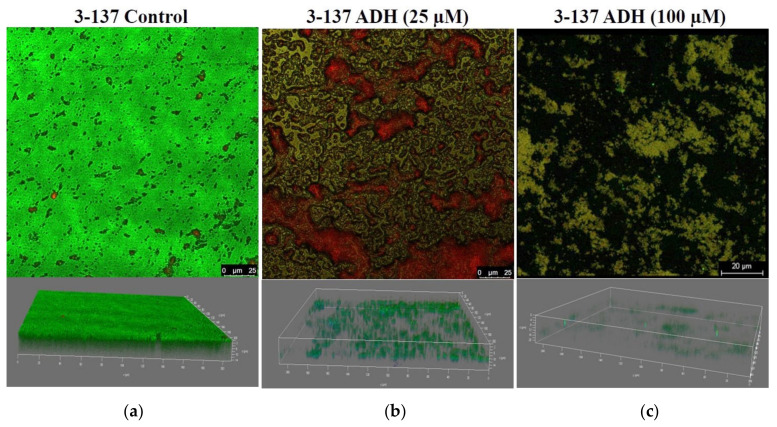
CLSM imaging of biofilm formation by strong biofilm former *A. baumannii* isolate 3-137 (**a**) control (untreated), (**b**) 48 h old biofilms treated with 25 µM, and (**c**) 100 µM ADH.

Confocal laser scanning microscopy was performed to validate the biofilm eradication potential of ADH. Three-dimensional images with only one channel (green) depicting the live cells are shown to give a glimpse of viable bacterial cells in the ADH-treated and untreated biofilms (Supplementary Figure S2). Since 25 µM of ADH was the lowest concentration showing significant biofilm eradication potential, and 100 µM demonstrated a significant reduction in biofilm-associated cfu/mL, these concentrations of ADH were analyzed for their potential to eliminate biofilms by confocal microscopy. Well-structured multi-layered biofilms were witnessed in the untreated control groups. By contrast, samples exposed to ADH (25 μM and 100 μM) post-biofilm formation showed not only the deterioration of biofilm structure but a drastic reduction in the biomass, surface area covered by the bacterial cells, and diminished thickness of biofilms, observed in a concentration-dependent manner (Figure 6).

### 3.6. Expression of Biofilm-Associated Genes Post-Biofilm Treatment with ADH

**Figure 7:**
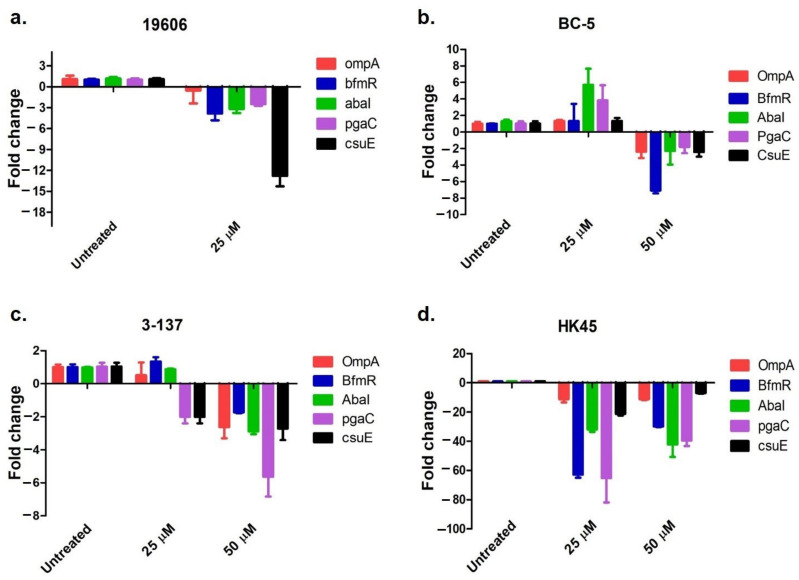
Expression of biofilm-associated genes (ompA, bfmR, abaI, pgaC, and csuE) in the control and on exposure to ADH at 8 MIC and 16 MIC of 48 h old biofilms formed by (**a**) ATCC 19606, (**b**) BC-5, (**c**) 3-137, and (**d**) HK45.

qRT-PCR was performed to analyze the expression of five biofilm-associated genes (ompA, bfmR, abaI, csuE, and pgaC) when exposed to ADH. The untreated culture was used as the control, and changes in the expression of biofilm-associated genes in biofilm-embedded cells exposed to 25 µM (8 × MIC of ADH) and 50 µM (16 × MIC of ADH) ADH concentrations were analyzed. All biofilm-associated genes tested were found to be downregulated at the 50 μM (16 × MIC) concentration in all the *A. baumannii* isolates (Figure 7). In the case of ATCC 19606 and HK-45, downregulation of biofilm-associated genes was observed on exposure to 25 μM concentrations of ADH (Figure 7a,d).

### 3.7. Combination of ADH along with Other Antibiotics for Synergy

A checkerboard assay was performed to determine the in vitro combination effects of ADH and other antibiotics for synergistic activity. The best among all was the combination of ADH and rifampicin with synergistic (∑FICI < 0.5) and additive activity (∑FICI = 0.5–1) of the ADH and rifampicin combination for 25% and 50% of *A. baumannii* isolates, followed by its combination with colistin, displaying additive activity against 50% of the tested *A. baumannii* isolates, with a FICI of 0.625. On the other hand, most of the antibiotic combinations exhibited indifferent activity (∑FICI = 1–4.0) against tested *A. baumannii* strains (Table 2).

## 4. Discussion

Bacterial biofilms are responsible for causing 80% of chronic infections in humans and pose difficulty in treatment due to the impermeable biofilm matrix, which restricts antibiotic access to biofilm-embedded cells [15,16]. Another theory explaining the resistance of biofilm to antibiotics is the dormant state of cells; most antibiotics target the synthesis of macromolecules, and cells in the dormant state lack metabolic activity, thus contributing to resistance to antibiotics [30]. Bacteria colonizing medical devices, such as ventilators, catheters, intravascular catheters, prosthetic joints, and cardiac devices, can spread to patient tissues and pose a severe health risk. Decontaminating medical devices is an effective way to control the spread of infections in healthcare centers.

To achieve the current requirements of novel antibiotic and antibiofilm compounds against *A. baumannii*, in this study, we screened 400 chemically distinct compounds provided by MMV as a chemical library contained in the Pandemic Response Box. The antimicrobial susceptibility of these compounds against *A. baumannii* was determined using a resazurin-based broth microdilution method, reliable for high throughput screening, as depicted by the Z-factor of 0.904. Screening wide-range compounds is easy with this method, as it provides precise results when screening colored compounds, including biosurfactants [22]. In the past, this assay has been utilized to screen for polymixin resistance in *A. baumannii* and for high-throughput screenings of *Mycobacterium chimeras* [21,31]. We identified 30 compounds (3 antifungal and 27 antibacterial) with potential antibacterial activity at ≤50 μM concentrations against planktonic *A. baumannii* cells. Of the 27 antibacterial compounds, 11 were already well-known, established antibacterial compounds. Among these 30 compounds, 21 exhibited growth inhibition of the *A. baumannii* reference isolates, ATCC 19606 and BC-5. A total of 12 (1 antifungal and 11 antibacterial) of these 21 compounds showing antibacterial activity against both the isolates at MIC ≤25 μM were further analyzed for their anti-biofilm properties. We identified five potential compounds that showed a reduction in the metabolic activity of biofilm-associated cells. Two of these five compounds, eravacycline and sitafloxacin, are already known for their antibacterial activity against *A. baumannii* [32,33,34,35].

Eravacycline exhibited the lowest MIC of 0.78 μM among all the compounds tested and showed potential antibiofilm activity against *A. baumannii*. Eravacycline (MMV1578574) is already known for its activity against carbapenem-resistant *A. baumannii*, and there are also reports of eravacycline resistance among Gram-negative bacteria [36,37,38]. In this study, Gepotidacin (MMV1578568) and sitafloxacin (MMV1579850) exhibited higher (25 µM) and varying MIC values for ATCC 19606 and BC-5, and since a higher concentration of antibiotic is required for targeting biofilm-associated cells than planktonic cells, gepotidacin and sitafloxacin were excluded from the study. Of the five compounds with antibiofilm properties against *A. baumannii* 19606 and BC-5, a compound with the MMV ID MMV396785, alexidine dihydrochloride (ADH), was selected for further analysis due to its low MIC and a wide range of actions [39]. Another compound, MUT056399 (MMV1578564), showed similar results as ADH against *A. baumannii* biofilms.

ADH is a bisbiguanide compound known for its antimicrobial properties. It is being used as an anti-plaque agent in oral disinfectants and lens cleansers [40,41,42]. Previous studies have shown the antifungal activity of ADH against *Candida albicans*, *Candida auris*, *Cryptococcus neoformans*, and *Aspergillus fumigatus* in the planktonic and biofilm phases. A study showed in vivo biofilm eradication by ADH in a mouse central venous catheter at concentrations lower than those cytotoxic to mammalian cells [39]. Another in vivo study showed that the topical application of alexidine at 100 mg/L concentration to Chinese hamster cornea is less toxic than chlorhexidine [43]. ADH showed antifungal activity when applied topically to diabetic mice infected with *Trichophyton mentagrophytes* [44]. In another study, where ADH was tested for antifungal activity against the pathogen *Cryptococcus neoformans*, it selectively inhibited cytosolic phospholipase B [45]. ADH was also identified as a novel anticancer therapeutic for head and neck cancer as it induces mitochondrial damage by targeting protein tyrosine phosphatase and, consequently, cell apoptosis [46]. Zorko et al., 2008 also showed the potential antibacterial and antibiofilm activity of ADH against *E. coli* and *S. aureus*. Further, their study also suggested the use of bisbiguanide for topical application along with other antibiotics, which will help suppress the proinflammatory response occurring due to bacterial killing by antibiotics [47]. Since ADH is positively charged, it might bind to the negatively charged membranes of *A. baumannii*, causing membrane perturbation and, ultimately, cell death [48,49]. In concordance with the above studies, our results showed ADH’s potential antibacterial and antibiofilm activity against *A. baumannii* isolates. The bactericidal activity of ADH demonstrated by the time–kill assay is advantageous as it is hypothesized that antimicrobial agents with bactericidal activity display better clinical outcomes with a speedy recovery [50]. We determined the minimum biofilm inhibition concentration (MBIC) and minimum biofilm eradication concentration (MBEC) of ADH to be 12.5 μM and 25–100 μM, respectively, using a crystal violet assay. The compound inhibited biofilm formation by ≥93% and eradicated pre-formed biofilms by 60–77.4% at 12.5 μM and 25–100 μM concentrations, respectively. When combined with rifampicin and colistin, the bisbiguanide compound demonstrated an additive antibacterial effect on 75% and 50% of isolates, respectively, making it a viable option for combination therapy. The broad range of antibiofilm activity of ADH against fungal and bacterial pathogens will serve as a guide for its application to control infections in medical centers.

Predominantly, exposure to sub-inhibitory antibiotic concentrations induces bacterial cells to switch to a biofilm mode of lifestyle to enable protection [51,52]. We examined the expression of biofilm-associated genes *ompA, pgaC, csuE, bfmR,* and *abaI* in *A. baumannii* following exposure to ADH below MBEC. Our results showed downregulation of all biofilm-associated genes, including genes playing a role in adhesion (*ompA* and *csuE*) matrix formation (*pgaC*), and biofilm regulation (*bfmR* and *abaI*) at 25 μM for isolates 19606 and HK-45 and 50 μM for isolates BC-5 and 3-137 when compared to the untreated controls. BfmR, the regulator component of the two-component system BfmRS, plays a crucial role in biofilm formation by regulating the expression of the *csu* operon. Our results are in concordance with this previous observation showing synchronized downregulation of *bfmR* and *csuE* [53]. A recent study identified the role of LeuO, a LysR-type transcription regulator that regulates the expression of genes involved in biofilm formation. LeuO mutant *A. baumannii* isolates produced more biofilms, and genes, including *abaI* and the *csu* operon, were found to be upregulated [54]. Studying the effects of ADH on transcription factors such as LeuO will contribute to understanding the molecular mechanisms governing biofilm formation and developing strategies for controlling *A. baumannii* biofilm-related infections.

Currently, medical-device-associated infections account for more than half of nosocomial infections resulting in massive losses in terms of life and economy [30,55]. The most acceptable way of controlling the spread of infections is to prevent bacterial attachment since pre-formed biofilms are tolerant to antibiotics or require much higher concentrations of antibiotics for complete clearance. Inhibition of biofilm formation can be achieved by coating medical devices with appropriate combinations of antibiotics and preventing colonization by bacterial cells. Due to its ability to substantially eradicate pre-formed biofilms and inhibit colonization by bacterial cells, ADH is an appropriate option for this task.

## Figures and Tables

**Table 1:** List of biofilm-associated genes with their role and primer sequences analyzed in this study.

S. No.	Gene	Role	Primer Sequence	Amplicon Size
1	*ompA*	Invasion, serum resistance, and biofilm formation	F-CGCAGCTCTTGGTATCGAGT R-CGGCTTGATTTTGCTGTCGT	177
2	*csuE*	Formation of pilus structure and initial adherence for biofilm formation	F-TGAGCTAAAATTCGGCAGTC R-TCTTTGAGAGTCCTGGGTTT	121
3	*pgaC*	*N*-glycosyltransferase, synthesis of PNAG	F-TATGTGGCCGGTAATGCTCG R-TATCACGCCATACCACTGCG	151
4	*bfmR*	Response regulator of two-component system bfmRS associated with biofilm formation	F-ATTCGTGCTTTGTTACGCCG R-GCGATAAAATACGGCCAGCG	190
5	*abaI*	Synthesis of quorum-sensing molecule, AHL	F-CCCGCAGCACGTAATAAACG R-AGCAGTCAGGCTGTGTCATC	134
6	*16S rRNA*	Endogenous control	F-ACTTTAAGCGAGGAGGAGGC R-GATTAACGCTCGCACCCTCT	123
7	*rpoB*	Endogenous control	F-TCCTTGAACACGATGACGCA R-GCAACGTTCGCTTCCATACC	118

**Table 2:** Results of checkerboard assay for ADH in combination with tigecycline, rifampicin, colistin, and trimethoprim against *A. baumannii* strains.

S. No.	Antibiotic with ADH	Strain	FICI	Effect
1	Tigecycline	19606	0.75	Additive
		BC-5	1.25	Indifferent
		3-137	1.25	Indifferent
		HK-45	1	Indifferent
2	Rifampicin	19606	1.125	Indifferent
		BC-5	0.625	Additive
		3-137	0.5625	Additive
		HK45	0.3125	Synergistic
3	Trimethoprim	19606	0.75	Additive
		BC-5	0.75	Additive
		3-137	1.125	Indifferent
		HK45	1.03125	Indifferent
4	Colistin	19606	1	Indifferent
		BC-5	0.625	Additive
		3-137	1.03125	Indifferent
		HK-45	0.625	Additive

## Data Availability

All the data are available in the manuscript.

## Supplementary Materials

The following supporting information can be downloaded at: https://www.mdpi.com/article/10.3390/antibiotics12071155/s1, Figure S1: Biofilm eradication potential of twelve compounds against *Acinetobacter baumannii* ATCC 19606 and BC-5 isolates. Figure S2: CLSM images displaying biofilm eradication by Alexidine dihydrochloride at 25 μM and 100 μM in all tested *A. baumannii* isolates. Table S1: MICs of 30 compounds from the MMV Pandemic Response Box displaying antibacterial properties against *A. baumannii.*

## References

[1] WHO Publishes List of Bacteria for Which New Antibiotics Are Urgently Needed. https://www.who.int/news/item/27-02-2017-who-publishes-list-of-bacteria-for-which-new-antibiotics-are-urgently-needed.

[2] Zhou M., Wang H., Zeng X., Yin P., Zhu J., Chen W., Li X., Wang L., Wang L., Liu Y. (2019). Mortality, morbidity, and risk factors in China and its provinces, 1990-2017: A systematic analysis for the Global Burden of Disease Study 2017. Lancet.

[3] Collaborators A.R. (2022). Global burden of bacterial antimicrobial resistance in 2019: A systematic analysis. Lancet.

[4] Fournier P.E., Richet H. (2006). The epidemiology and control of *Acinetobacter baumannii* in health care facilities. Clin. Infect. Dis..

[5] Lin M.F., Lan C.Y. (2014). Antimicrobial resistance in *Acinetobacter baumannii*: From bench to bedside. World J. Clin. Cases.

[6] Chang H.C., Chen Y.C., Lin M.C., Liu S.F., Chung Y.H., Su M.C., Fang W.F., Tseng C.C., Lie C.H., Huang K.T. (2011). Mortality risk factors in patients with *Acinetobacter baumannii* ventilator: Associated pneumonia. J. Formos. Med. Assoc..

[7] Cornejo-Juárez P., Cevallos M.A., Castro-Jaimes S., Castillo-Ramírez S., Velázquez-Acosta C., Martínez-Oliva D., Pérez-Oseguera A., Rivera-Buendía F., Volkow-Fernández P. (2020). High mortality in an outbreak of multidrug resistant *Acinetobacter baumannii* infection introduced to an oncological hospital by a patient transferred from a general hospital. PLoS ONE.

[8] John A.O., Paul H., Vijayakumar S., Anandan S., Sudarsan T., Abraham O.C., Balaji V. (2020). Mortality from *Acinetobacter* infections as compared to other infections among critically ill patients in South India: A prospective cohort study. Indian J. Med. Microbiol..

[9] Vivo A., Fitzpatrick M.A., Suda K.J., Jones M.M., Perencevich E.N., Rubin M.A., Ramanathan S., Wilson G.M., Evans M.E., Evans C.T. (2022). Epidemiology and outcomes associated with carbapenem-resistant *Acinetobacter baumannii* and carbapenem-resistant *Pseudomonas aeruginosa*: A retrospective cohort study. BMC Infect. Dis..

[10] Alrahmany D., Omar A.F., Alreesi A., Harb G., Ghazi I.M. (2022). *Acinetobacter baumannii* Infection-Related Mortality in Hospitalized Patients: Risk Factors and Potential Targets for Clinical and Antimicrobial Stewardship Interventions. Antibiotics.

[11] Rodríguez-Baño J., Martí S., Soto S., Fernández-Cuenca F., Cisneros J.M., Pachón J., Pascual A., Martínez-Martínez L., McQueary C., Actis L.A. (2008). Biofilm formation in *Acinetobacter baumannii*: Associated features and clinical implications. Clin. Microbiol. Infect..

[12] Poorzargar P., Javadpour S., Karmostaji A. (2017). Distribution and antibiogram pattern of *Acinetobacter* infections in Shahid Mohammadi Hospital, Bandar Abbas, Iran. Bimon. J. Hormozgan Univ. Med. Sci..

[13] Colquhoun J.M., Rather P.N. (2020). Insights Into Mechanisms of Biofilm Formation in *Acinetobacter baumannii* and implications for uropathogenesis. Front. Cell. Infect. Microbiol..

[14] Upmanyu K., Haq Q.M.R., Singh R. (2022). Factors mediating *Acinetobacter baumannii* biofilm formation: Opportunities for developing therapeutics. Curr. Res. Microb. Sci..

[15] Eze E.C., Chenia H.Y., El Zowalaty M.E. (2018). Acinetobacter baumannii biofilms: Effects of physicochemical factors, virulence, antibiotic resistance determinants, gene regulation, and future antimicrobial treatments. Infect. Drug Resist..

[16] Gayoso C.M., Mateos J., Méndez J.A., Fernández-Puente P., Rumbo C., Tomás M., Martínez de Ilarduya O., Bou G. (2014). Molecular mechanisms involved in the response to desiccation stress and persistence in *Acinetobacter baumannii*. J. Proteome Res..

[17] Shenkutie A.M., Yao M.Z., Siu G.K., Wong B.K.C., Leung P.H. (2020). Biofilm-induced antibiotic resistance in clinical *Acinetobacter baumannii* isolates. Antibiotics.

[18] Roy S., Chowdhury G., Mukhopadhyay A.K., Dutta S., Basu S. (2022). Convergence of biofilm formation and antibiotic resistance in *Acinetobacter baumannii* infection. Front. Med..

[19] Clinical and Laboratory Standards Institute (2022). Performance Standards for Antimicrobial Susceptibility Testing.

[20] Raorane C.J., Lee J.-H., Lee J. (2020). Rapid Killing and Biofilm Inhibition of Multidrug-Resistant *Acinetobacter baumannii* Strains and Other Microbes by Iodoindoles. Biomolecules.

[21] Cantillon D., Goff A., Taylor S., Salehi E., Fidler K., Stoneham S., Waddell S.J. (2022). Searching for new therapeutic options for the uncommon pathogen *Mycobacterium chimaera*: An open drug discovery approach. Lancet Microbe.

[22] Elshikh M., Ahmed S., Funston S., Dunlop P., McGaw M., Marchant R., Banat I.M. (2016). Resazurin-based 96-well plate microdilution method for the determination of minimum inhibitory concentration of biosurfactants. Biotechnol. Lett..

[23] Soudeiha M.A.H., Dahdouh E.A., Azar E., Sarkis D.K., Daoud Z. (2017). In vitro evaluation of the colistin-carbapenem combination in clinical isolates of *A. baumannii* using the checkerboard, E-test, and time-kill curve Techniques. Front. Cell. Infect. Microbiol..

[24] Haney E.F., Trimble M.J., Cheng J.T., Vallé Q., Hancock R.E.W. (2018). Critical Assessment of Methods to Quantify Biofilm Growth and Evaluate Antibiofilm Activity of Host Defence Peptides. Biomolecules.

[25] Di Somma A., Recupido F., Cirillo A., Romano A., Romanelli A., Caserta S., Guido S., Duilio A. (2020). Antibiofilm properties of Temporin-L on *Pseudomonas fluorescens* in static and in-flow conditions. Int. J. Mol. Sci..

[26] Ding Y., Wang W., Fan M., Tong Z., Kuang R., Jiang W., Ni L. (2014). Antimicrobial and anti-biofilm effect of Bac8c on major bacteria associated with dental caries and *Streptococcus mutans* biofilms. Peptides.

[27] Thamlikitkul V., Tiengrim S. (2014). In vitro activity of colistin plus sulbactam against extensive-drug-resistant *Acinetobacter baumannii* by checkerboard method. J. Med. Assoc. Thail..

[28] Zhang J.H., Chung T.D., Oldenburg K.R. (1999). A Simple statistical parameter for use in evaluation and validation of high throughput screening assays. J. Biomol. Screen..

[29] Mathur H., Field D., Rea M.C., Cotter P.D., Hill C., Ross R.P. (2018). Fighting biofilms with lantibiotics and other groups of bacteriocins. NPJ Biofilms Microbiomes.

[30] Mi G., Shi D., Wang M., Webster T.J. (2018). Reducing bacterial infections and biofilm formation using nanoparticles and nanostructured antibacterial surfaces. Adv. Healthc. Mater..

[31] Lescat M., Poirel L., Tinguely C., Nordmann P. (2019). A Resazurin Reduction-Based Assay for Rapid Detection of Polymyxin Resistance in Acinetobacter baumannii and Pseudomonas aeruginosa. J. Clin. Microbiol..

[32] Abdallah M., Olafisoye O., Cortes C., Urban C., Landman D., Quale J. (2015). Activity of eravacycline against Enterobacteriaceae and *Acinetobacter baumannii*, including multidrug-resistant isolates, from New York City. Antimicrob. Agents Chemother..

[33] Livermore D.M., Mushtaq S., Warner M., Woodford N. (2016). In vitro activity of eravacycline against carbapenem-resistant Enterobacteriaceae and *Acinetobacter baumannii*. Antimicrob. Agents Chemother..

[34] Alosaimy S., Morrisette T., Lagnf A.M., Rojas L.M., King M.A., Pullinger B.M., Hobbs A.L.V., Perkins N.B., Veve M.P., Bouchard J. (2022). Clinical outcomes of eravacycline in patients treated predominately for carbapenem-resistant *Acinetobacter baumannii*. Microbiol. Spectr..

[35] Rodjun V., Houngsaitong J., Montakantikul P., Paiboonvong T., Khuntayaporn P., Yanyongchaikit P., Sriyant P. (2020). In vitro activities of colistin and sitafloxacin combinations against multidrug-, carbapenem-, and colistin-resistant *Acinetobacter baumannii* using the broth microdilution checkerboard and time-kill methods. Antibiotics.

[36] Wang L., Liu D., Lv Y., Cui L., Li Y., Li T., Song H., Hao Y., Shen J., Wang Y. (2019). Novel plasmid-mediated tet(x5) gene conferring resistance to tigecycline, eravacycline, and omadacycline in a clinical *Acinetobacter baumannii* isolate. Antimicrob. Agents Chemother..

[37] Fyfe C., LeBlanc G., Close B., Nordmann P., Dumas J., Grossman T.H. (2016). Eravacycline is active against bacterial isolates expressing the polymyxin resistance gene *mcr-1*. Antimicrob. Agents Chemother..

[38] Shi Y., Hua X., Xu Q., Yang Y., Zhang L., He J., Mu X., Hu L., Leptihn S., Yu Y. (2020). Mechanism of eravacycline resistance in *Acinetobacter baumannii* mediated by a deletion mutation in the sensor kinase adeS, leading to elevated expression of the efflux pump AdeABC. Infect. Genet. Evol..

[39] Mamouei Z., Alqarihi A., Singh S., Xu S., Mansour M.K., Ibrahim A.S., Uppuluri P. (2018). Alexidine dihydrochloride has broad-spectrum activities against diverse fungal pathogens. mSphere.

[40] Bonesvoll P., Gjermo P. (1978). A comparision between chlorhexidine and some quaternary ammonium compounds with regard to retention, salivary concentration and plaque-inhibiting effect in the human mouth after mouth rinses. Arch. Oral. Biol..

[41] Lobene R.R., Soparkar P.M. (1973). The effect of an alexidine mouthwash on human plaque and gingivitis. J. Am. Dent. Assoc..

[42] Barnes G.P., Carter H.G., Gross A., Bhaskar S.N., Schildt N.N., Bush A.G. (1972). Dental plaque reduction with an antibacterial mouth rinse. Part I. Oral Surg. Oral Med. Oral Pathol..

[43] Alizadeh H., Neelam S., Cavanagh H.D. (2009). Amoebicidal activities of alexidine against 3 pathogenic strains of acanthamoeba. Eye Contact Lens.

[44] Nabeela S., Date A., Ibrahim A.S., Uppuluri P. (2022). Antifungal activity of alexidine dihydrochloride in a novel diabetic mouse model of dermatophytosis. Front. Cell. Infect. Microbiol..

[45] Shea J.M., Henry J.L., Del Poeta M. (2006). Lipid metabolism in *Cryptococcus neoformans*. FEMS Yeast Res..

[46] Yip K.W., Ito E., Mao X., Au P.Y., Hedley D.W., Mocanu J.D., Bastianutto C., Schimmer A., Liu F.F. (2006). Potential use of alexidine dihydrochloride as an apoptosis-promoting anticancer agent. Mol. Cancer Ther..

[47] Zorko M., Jerala R. (2008). Alexidine and chlorhexidine bind to lipopolysaccharide and lipoteichoic acid and prevent cell activation by antibiotics. J. Antimicrob. Chemother..

[48] Thangavelu A., Kaspar S.S., Kathirvelu R.P., Srinivasan B., Srinivasan S., Sundram R. (2020). Chlorhexidine: An elixir for periodontics. J. Pharm. Bioallied Sci..

[49] Houston R., Sekine Y., Larsen M.B., Murakami K., Mullett S.J., Wendell S.G., Narendra D.P., Chen B.B., Sekine S. (2021). Discovery of bactericides as an acute mitochondrial membrane damage inducer. Mol. Biol. Cell.

[50] Alder J., Eisenstein B. (2004). The advantage of bactericidal drugs in the treatment of infection. Curr. Infect. Dis. Rep..

[51] Kaplan J.B. (2011). Antibiotic-induced biofilm formation. Int. J. Artif. Organs.

[52] Penesyan A., Paulsen I.T., Gillings M.R., Kjelleberg S., Manefield M.J. (2020). Secondary effects of antibiotics on microbial biofilms. Front. Microbiol..

[53] Gao Q., Meng X., Gu H., Chen X., Yang H., Qiao Y., Guo X. (2019). Two Phenotype-Differentiated *Acinetobacter baumannii* mutants that survived in a meropenem selection display large differences in their transcription profiles. Front. Microbiol..

[54] Islam M.M., Kim K., Lee J.C., Shin M. (2021). LeuO, a LysR-type transcriptional regulator, is involved in biofilm formation and virulence of *Acinetobacter baumannii*. Front. Cell. Infect. Microbiol..

[55] Wang W., Chanda W., Zhong M. (2015). The relationship between biofilm and outer membrane vesicles: A novel therapy overview. FEMS Microbiol. Lett..

