# Supplementary material for: Antibacterial and Antibiofilm Properties of the Alexidine Dihydrochloride (MMV396785) against Acinetobacter baumannii
Source: Antibiotics (Basel). 2023 Jul 6;12(7):1155. doi: 10.3390/antibiotics12071155 (PMC10375957; doi:10.3390/antibiotics12071155)
Supplement: Supplementary file 1 [file antibiotics-12-01155-s001.zip › Supplementary Table S1.pdf]

Supplementary Table S1: MIC of 30 compounds from MMV pandemic response box displaying antibacterial properties against *A. baumannii*

S. No.	MMV ID	Chemical Name / (Trivial Name)	Molecular Weight	MIC (µM) against ATCC 19606	MIC (µM) against BC-5
1.	MMV687273	N'-(2-adamantyl)-N-[(2E)-3,7-dimethylocta-2,6-dienyl]ethane-1,2-diamine / (SQ109)	330.6	50	50
2.	MMV1581548	2-(3-tert-butyl-2-hydroxyphenyl)-1H-benzimidazole-5-carboximidamide	308.4	50	50
3.	MMV1578558	(2-ethyl-1-benzofuran-3-yl)-(4-hydroxyphenyl)methanone/ (Benzarone)	266.3	>50	50
4.	MMV1480967	1-cyclopropyl-6,7-difluoro-8-methoxy-4-oxoquinoline-3-carboxylic acid	295.2	25	50
5.	MMV1578564	4-(4-ethyl-5-fluoro-2-hydroxyphenoxy)-3-fluorobenzamide / (MUT056399)	293.3	6.25	6.25
6.	MMV396785	1-(2-ethylhexyl)-3-[N-[6-[[N-[N-(2-ethylhexyl)carbamimidoyl]carbamimidoyl]amino]hexyl]carbamimidoyl]guanidine / (Alexidine)	581.72	3.125	3.125
7.	MMV000051	rac-(2S,4R)-1-methyl-4-propyl-N-[rac-(1S,2S)-2-chloro-1-[rac-(2R,3R,4S,5R,6R)-3,4,5-trihydroxy-6-methylsulfanyloxan-2-yl]propyl]pyrrolidine-2-carboxamide / (Clindamycin)	461.46	50	50
8.	MMV1633674	[(1S,2R,3S,4S,6R,7R,8R,14R)-4-ethenyl-3-hydroxy-2,4,7,14-tetramethyl-9-oxo-6-tricyclo[5.4.3.01,8]tetradecanyl] 2-[[(1S,5R)-8-methyl-8-azabicyclo[3.2.1]octan-3-yl]sulfanyl]acetate / (Retapamulin)	517.8	50	>50
9.	MMV1782140	N-[5-(4-chlorophenyl)-1,3,4-oxadiazol-2-yl]-4-fluorobenzamide	317.7	>50	50
10.	MMV637659	7-fluoro-8-(4-hydroxypiperidin-1-yl)-12-methyl-4-oxo-1-azatricyclo[7.3.1.05,13]trideca-2,5,7,9(13)-tetraene-3-carboxylic acid / (Nadifloxacin)	360.4	>50	50
11.	MMV002224	3-benzamidopropanoic acid / (Betamipron)	193.2	50	>50
12.	MMV000043	4-N-[2,6-dimethoxy-4-methyl-5-[3-(trifluoromethyl)phenoxy]quinolin-8-yl]pentane-1,4-diamine / (Tafenoquine)	581.6	25	50
13.	MMV002459	(2R,3S,4S,5R,6S)-4-amino-2-(hydroxymethyl)-6-[(1S,2S,3R,4S,6R)-4,6-diamino-2-hydroxy-3-[(2R,3R,5S,6R)-3-amino-6-(aminomethyl)-5-hydroxyoxan-2-yl]oxycyclohexyl]oxyoxane-3,5-diol / (Tobramycin)	467.5	25	50
14.	MMV637945	(2S,5R)-3,3-dimethyl-4,4,7-trioxo-4?6-thia-1-azabicyclo[3.2.0]heptane-2-carboxylic acid / (Sulbactam)	233.2	25	>50
15.	MMV1578570	1-(butan-2-ylamino)-3-(3,6-dichlorocarbazol-9-yl)propan-2-ol	365.3	50	50
16.	MMV000725	1-(2,4-dichlorophenoxy)-3-[3-[2-(diethylamino)ethyl]-2-iminobenzimidazol-1-yl]propan-2-ol	451.4	25	>50
17.	MMV1634399	4-methyl-8-phenoxy-1-(2-phenylethyl)-2,3-dihydropyrrolo[3,2-c]quinoline	380.5	50	>50

18.	MMV1578568	3-[[4-(3,4-dihydro-2H-pyrano[2,3-c]pyridin-6-ylmethylamino)piperidin-1-yl]methyl]-1,4,7-triazatricyclo[6.3.1.04,12]dodeca-6,8(12),9-triene-5,11-dione	448.5	25	12.5
19.	MMV1593541	6-N-(2-amino-6-phenylpyrimidin-4-yl)-2-(4-fluorophenyl)quinoline-4,6-diamine	422.5	12.5	25
20.	MMV003137	rac-(3R,4S,5S,6R,7R,9R,11R,12R,13S,14R)-14-ethyl-7,12,13-trihydroxy-3,5,7,9,11,13-hexamethyl-6-[rac-(2S,3R,4S,6R)-4-(dimethylamino)-3-hydroxy-6-methyloxan-2-yl]oxy-4-[rac-(2R,4R,5S,6S)-5-hydroxy-4-methoxy-4,6-dimethyloxan-2-yl]oxy-oxacyclotetradecane-2,10-dione **/** (Erythromycin)	733.9	>50	6.25
21.	MMV1580854	2-(2-aminopyridin-3-yl)oxy-5-ethyl-4-fluorophenol	248.3	6.25	12.5
22.	MMV1634402	4-N,6-N-bis[3-(5-carbamimidamidopentanoylamino)-2-[(3R)-pyrrolidin-3-yl]oxy-5-(trifluoromethyl)phenyl]pyrimidine-4,6-dicarboxamide	1621	25	12.5
23.	MMV1578566	3-[[(3S)-3-(aminomethyl)-1-hydroxy-3H-2,1-benzoxaborol-7-yl]oxy]propan-1-ol **/** (Epetraborole)	273.56	6.25	6.25
24.	MMV1579850	7-(7-amino-5-azaspiro[2.4]heptan-5-yl)-8-chloro-6-fluoro-4-oxo-1-[rac-(1R,2S)-2-fluorocyclopropyl]quinoline-3-carboxylic acid **/** (Sitafloxacin)	436.84	1.562	25
25.	MMV1579780	2-N,6-N-dibenzyl-9-propan-2-ylpurine-2,6-diamine	372.5	3.125	25
26.	MMV1580173	Trimetrexate	369.4	6.25	6.25
27.	MMV002731	6-cyclohexyl-1-hydroxy-4-methylpyridin-2-one **/** (Ciclopirox)	207.3	50	50
28.	MMV002350	1-ethyl-6,8-difluoro-7-(3-methylpiperazin-1-yl)-4-oxoquinoline-3-carboxylic acid **/** (Lomefloxacin)	387.86	6.25	50
29.	MMV1578574	(4S,4aS,5aR,12aR)-4-(dimethylamino)-7-fluoro-1,10,11,12a-tetrahydroxy-3,12-dioxo-9-[(2-pyrrolidin-1-ylacetyl)amino]-4a,5,5a,6-tetrahydro-4H-tetracene-2-carboxamide **/** (Eravacycline)	631.52	0.78	0.78
30	MMV1578554	3,4-dibromopyrrole-2,5-dione **/** (2,3-Dibromomaleinimide)	254.9	12.5	25
