# Supplementary figures and images for: Antibacterial and Antibiofilm Properties of the Alexidine Dihydrochloride (MMV396785) against Acinetobacter baumannii
Source: Antibiotics (Basel). 2023 Jul 6;12(7):1155. doi: 10.3390/antibiotics12071155 (PMC10375957; doi:10.3390/antibiotics12071155)

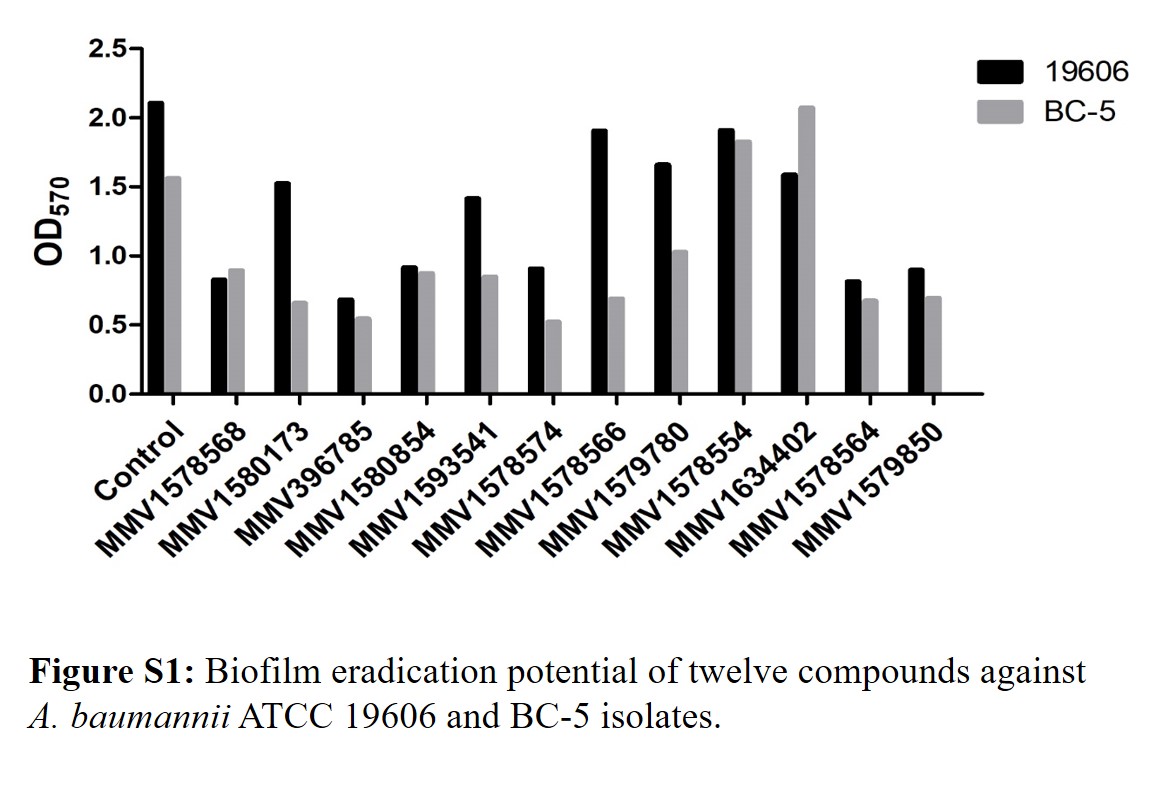

Supplement: Supplementary file 1 [file antibiotics-12-01155-s001.zip › Figure S1_MIC of 30 compounds.jpg]

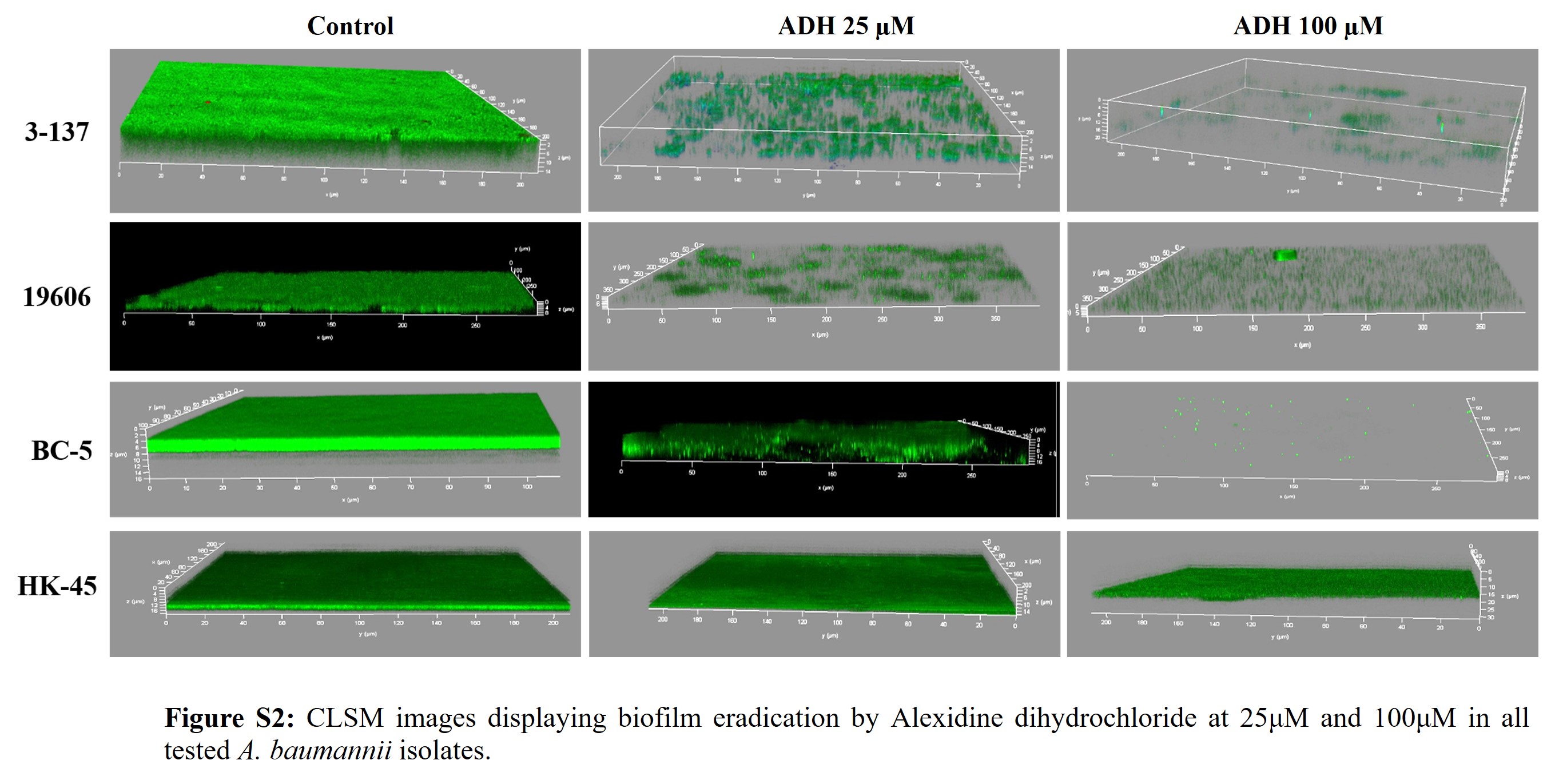

Supplement: Supplementary file 1 [file antibiotics-12-01155-s001.zip › Figure S2.jpg]
